# Identification of a Chimera Mass Spectrum of Isomeric Lipid A Species Using Negative Ion Tandem Mass Spectrometry

**DOI:** 10.3390/toxins16070322

**Published:** 2024-07-18

**Authors:** Ágnes Dörnyei, Anikó Kilár, Viktor Sándor

**Affiliations:** 1 Department of Analytical and Environmental Chemistry and Szentágothai Research Centre, Faculty of Sciences, University of Pécs, Ifjúság útja 6., H-7624 Pécs, Hungary; 2 Institute of Bioanalysis, Medical School, University of Pécs, Szigeti út 12., H-7624 Pécs, Hungary; aniko.kilar@aok.pte.hu (A.K.); sandor.viktor@pte.hu (V.S.)

**Keywords:** bacterial endotoxin, lipid A, constitutional isomers, negative ionization mode, energy-resolved tandem mass spectrometry, fragmentation pathways, structure elucidation

## Abstract

The toxic nature of bacterial endotoxins is affected by the structural details of lipid A, including the variety and position of acyl chains and phosphate group(s) on its diglucosamine backbone. Negative-ion mode tandem mass spectrometry is a primary method for the structure elucidation of lipid A, used independently or in combination with separation techniques. However, it is challenging to accurately characterize constitutional isomers of lipid A extracts by direct mass spectrometry, as the elemental composition and molecular mass of these molecules are identical. Thus, their simultaneous fragmentation leads to a composite, so-called chimera mass spectrum. The present study focuses on the phosphopositional isomers of the classical monophosphorylated, hexaacylated *Escherichia coli*-type lipid A. Collision-induced dissociation (CID) was performed in an HPLC-ESI-QTOF system. Energy-resolved mass spectrometry (ERMS) was applied to uncover the distinct fragmentation profiles of the phosphorylation isomers. A fragmentation strategy applying multi-levels of collision energy has been proposed and applied to reveal sample complexity, whether it contains only a 4′-phosphorylated species or a mixture of 1- and 4′-phosphorylated variants. This comparative fragmentation study of isomeric lipid A species demonstrates the high potential of ERMS-derived information for the successful discrimination of co-ionized phosphorylation isomers of hexaacylated lipid A.

## 1. Introduction

Gram-negative bacteria are responsible for a variety of foodborne diseases and healthcare-associated infections. The outer leaflet of their outer membrane is composed mainly of lipopolysaccharides (LPSs). The beneficial (immunostimulatory) and adverse (e.g., pro-inflammatory) effects of LPS molecules released from the surface of bacteria depend largely on the structure of lipid A, the lipophilic anchor constituent of LPSs [1]. Lipid A, also known as endotoxin, can trigger the mammalian innate immune system through the Toll-like receptor 4 (TLR4)/MD-2 complex [2]. TLR4 is important for the clearance of infections; nevertheless, its overstimulation can ultimately lead to lethal septic shock, namely endotoxin shock.

The lipid A moiety typically consists of a diglucosamine backbone flanked by terminal phosphate groups and ester- and amide-linked fatty acyl chains [3]. The maximum stimulatory effect of the TLR4/MD2 complex is evoked by the bisphosphorylated, hexa-acylated lipid A molecule synthetized by *Escherichia coli* [4]. It consists of a β-1′,6-linked glucosamine disaccharide phosphorylated at the 1 and 4′ positions and acylated with four 3-hydroxymyristic acids at the 2, 3, 2′, and 3′ positions, of which those at the 2′ and 3′ positions are further acylated with lauric acid and myristic acid through their 3-hydroxyl groups. Variations in the fatty acyl composition or the number of phosphate groups can alter the strength of the TLR4/MD-2 response, reducing endotoxicity or even providing antagonistic activity to the lipid A molecule, and may also modify the sensitivity of bacteria to antibiotics [4,5,6,7]. Consequently, comprehensive characterization of the acylation and phosphorylation patterns of microbial lipid A samples is crucial in lipopolysaccharidomics studies, clinical diagnostics, and vaccine development experiments.

Tandem mass spectrometry is widely used for the structural characterization of bacterial lipid A molecules [8,9,10,11,12,13,14,15,16,17,18,19,20,21,22,23,24,25]. It can be used both as a standalone technique and in combination with various separation methods. However, one drawback is that in direct mass spectrometry measurements, or if the separation method fails to separate the constitutional isomers prior to tandem mass spectrometry, all isomeric species are simultaneously ionized in the mass spectrometer ion source and thus simultaneously selected for fragmentation. As a result, the MS/MS mass spectrum contains a mixture of fragment ions from several precursor molecules. This is called a chimera mass spectrum and is often characterized by a complex and overlapping peak pattern.

In the past few years, we have developed different methods combining tandem mass spectrometry with HPLC or non-aqueous CE to assess the natural heterogeneity of various lipid A samples [26,27,28,29,30]. Our analyses revealed that the bacterial samples contained several constitutional isomers of the monophosphorylated version of *E. coli*-type lipid A typical of *Enterobacteriaceae* strains. Based on detailed structural investigations, the constitutional isomers could be classified into two major groups: either the phosphate group was attached to the diglucosamine backbone in different positions (4′ or 1), or the constitution of the acyl chain region was different. However, phosphorylation isomers predominated; thus, for simplicity and clarity, we will refer to these isomers as P4′ and P1 hereafter. Since the majority of articles dealing with the mass spectrometric analysis of lipid A samples demonstrated only the structure of P4′ lipid As, and just a few published that of P1 ones, we believe that this variability (i.e., the co-presence of P4′ and P1 isomers) has probably remained hidden in previous direct MS/MS measurements [8,10,31,32,33]. Three possible reasons can be considered for the overrepresentation of P4′ species in the literature. (i) The examined lipid A samples of the bacterial isolates did not contain P1 species. (ii) The coexistence of the two phosphorylation isomers was not revealed because their chimera mass spectrum closely resembled the MS/MS mass spectrum of the predominant P4′ component, making it challenging and sometimes impossible to identify the minor P1 variant. Note that when analyzing isomeric ions, such as a mixture of phosphorylation isomers with identical acylation patterns, most of the fragment ions have the same elemental compositions and masses for both variants. (iii) Insufficient information on the fragmentation patterns of phosphorylation isomers led to incorrect conclusions about their acylation profiles instead of recognizing the phosphorylation difference (see an example of misidentification in [34]).

From thorough studies of the CID properties of deprotonated 4′-monophosphorylated lipid A precursor ions, the bond cleavage sequence of the acyl chains (at positions 3′ε > 3α > 3′α/β >> 2′ε) and the presence of A_2_-type cross-ring fragments in the spectrum are well known (for nomenclature see [35,36]). Meanwhile, a comprehensive description of the fragmentation attributes of deprotonated 1-monophosphorylated species is missing. Those few studies that either assumed the structures of deprotonated P1 lipid A species based on tandem mass spectrometry analysis [37,38] or partially addressed their fragmentation properties [12,14,28] contribute to our purpose of summarizing the peculiarities of the CID of such precursor ions, thereby providing a reliable toolbar for their recognition and structural characterization even in heterogeneous mixtures.

We aimed to investigate the energy-dependent fragmentation behavior of the deprotonated P4′ and P1 isomers of monophosphorylated *E. coli*-type lipid A species to find diagnostic ions to identify phosphorylation isomers from a chimera MS/MS mass spectrum. Additionally, we aimed to bring attention to the historical bias towards the *E. coli*-type P1 variants due to the limitations of direct MS/MS approaches and the unrevealed chimeric nature of the CID mass spectra found in the literature. Our final goal was to devise a strategy for identifying the chimeric nature of the CID mass spectra of lipid A species.

## 2. Results and Discussion

### 2.1. Energy Dependence of the Fatty Acyl Losses

The energy-resolved fragmentation of a 4′-monophosphorylated hexaacylated lipid A standard, i.e., PHAD-504, and its 1-monophosphorylated isomer (named P4′ and P1 variants) was studied by negative-ion tandem mass spectrometry. These lipid A molecules have a monoisotopic molecular mass of 1717.25306 u and differ only in the position of the phosphate group (at 4′ or 1) while having identical acylation patterns. The distribution of the six fatty acids on the backbone of both isomers is as follows: N2: C14:0(3-OH), O3: C14:0(3-OH), N2′: C14:0(3-O-C12:0), O3′: C14:0(3-O-C14:0). Both phosphorylation isomers are present in *E. coli* O83 strain. Currently, there is no commercially available P1 standard. Thus, we studied the P1 isomer after HPLC separation of our in-house standard mixture, i.e., an *E. coli* O83 lipid A extract. The extracted ion chromatogram for the separation of the P4′ and P1 isomers present in the natural extract and their chemical structures can be seen in Figure S1. The molar ratio of the P4′ and P1 isomers was approximately 85%:15% as deduced from their peak areas. The identity of the isomers was also proven by positive-ion MS/MS analysis (Figure S2).

**Figure 1:**
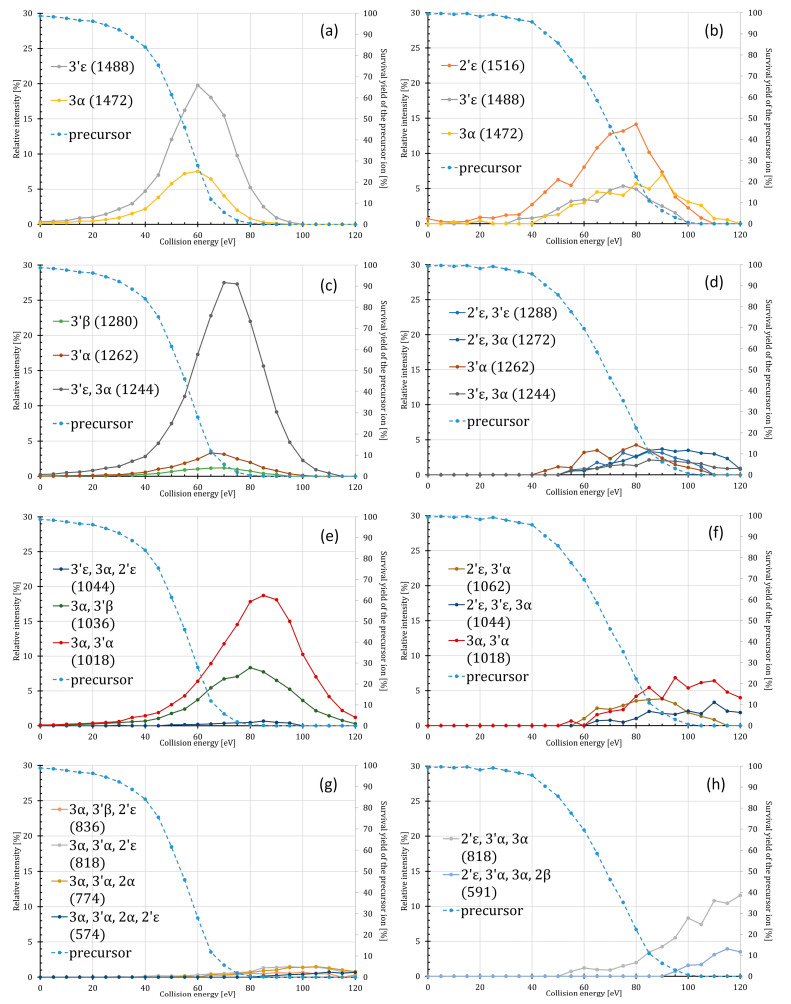
ERMS curves of the lipid A precursor ion, as well as fragment ions resulting from the consecutive and competitive losses of one (**a**,**b**), two (**c**,**d**), three (**e**,**f**), and four or five (**g**,**h**) fatty acyl chains. Diagrams on the left are for the P4′ isomer and on the right are for the P1 isomer. The numbers and Greek letters denote the cleavage sites and are also used to identify the fragment ions, the nominal ionic masses of which are indicated in parentheses. For exact masses of the ions and compositions of the neutral losses, see Table 1.

For both P1 and P4′ isomers, energy-resolved breakdown curves were constructed and compared, as shown in Figure 1 (the normalized breakdown curves can be found in Figure S3). (See a comparison of the ERMS graphs of P4′ present in the commercial standard, i.e., PHAD-504, and the in-house standard, i.e., an *E. coli* O83 extract, in Figure S4). For a better understanding of the ERMS profiles, we have collected the typical fatty acyl losses and fragment ion masses (see Table 1). The structures and cleavage sites of both isomers are presented in Figure S5. During the fragmentation of the deprotonated monophosphorylated lipid A species under CID conditions, predominantly the ester-bound fatty acyl chains are cleaved from the backbone in the order of their lability. Consequently, as the well-known classical *E. coli*-type acylation profile possesses high variability, the mass of the neutral losses (i.e., cleavable chains with different elemental composition) not only identifies the type of the fatty acyl chain (i.e., C12:0, C14:0, or C14:0(3-OH)), but also its position. The loss of the amide-linked acyl chains was detected only at high energies and never for the first generation of the fragments.

As seen in Figure 1, the product ion masses for the two isomers were essentially the same (with a few exceptions), which means that the fatty acyl cleavage sites are independent of the position of phosphorylation. Meanwhile, we observed that both the preference and energy dependence of the ester bond cleavages were different for the P4′ and P1 isomers (see discussion below). Such differences can greatly facilitate the identification and structural analysis of phosphorylation isomers using negative ion tandem mass spectrometry. In addition, there was also a difference in the degree of precursor fragmentation in the case of the P4′ and P1 isomers (Figure 1). The survival yield curves revealed that the P1 isomer exhibited greater stability compared to the P4′ isomer, as this variant required a CE of 68 eV to achieve SY_50%_, whereas the P4′ isomer required 53 eV (i.e., 15 eV less). Similarly, the steepness of the SY curves differed, with the P4′ precursor being completely decomposed at around 85 eV, while the more stable P1 required higher CE, approximately 105 eV, for complete decomposition.

The main features of the two isomer’s fragmentation are well illustrated by the breakdown diagrams (Figure 1). Specifically, the fragmentation process of the P4′ isomer (Figure 1a) starts with the competitive cleavages of the 3′ε and 3α bonds at low energies, resulting in fragment ions at *m*/*z* 1488 and 1472, respectively, where the 3′ε cleavage product is present in higher amount and in a slightly broader energy range than the 3α cleavage. Their intensity passes through a maximum of around 60 eV, where the SY of the P4′ precursor is *c.a.* 27%. At slightly higher CE, around 70 eV (where SY_P4′_ ≈ 6%), the product ion at *m*/*z* 1244 that formed from the combination of these two cleavages (3′ε with 3α) will dominate (Figure 1a). Besides this one, two other cleavage products appear to be formed by the 3′α and 3′β (i.e., at *m*/*z* 1262 and 1280, respectively), although with lower intensities and a slightly narrower energy range. The next generation of combined cleavages, such as 3α in combination with 3′α, and 3α with 3′β (at *m*/*z* 1018 and 1035), are more favored than any of the 3′α or 3′β cleavages alone (compare Figure 1c,e). Above 80 eV (when SY of the precursor reached 0%), cleavages in combination with the cleavage of the 2′ε or 2α bonds were minor or hardly detectable (giving product ions at *m*/*z* 836, 818, 774, and 574; see Figure 1g) resulting in a maximum of 1% or 2% relative intensities. Thus, we conclude from the ERMS analysis of the *E. coli*-type hexaacylated P4′ isomer that the fragmentation cleavage order is 3′ε > 3α > 3′α > 3′β >> 2′ε, which is in agreement with the literature data [8,10,26].

In the case of the P1 isomer, at low energies, the extent of 2′ε cleavage stands out from the others (3′ε, 3′α, 3α) (Figure 1b). In the low energy range (up to 80 eV, where SY_P1_ ≈ 20%), the ion at *m*/*z* 1516 is the most intense, and the other products (such as ions at *m*/*z* 1488, 1472, and 1262, corresponding to the 3′ε, 3α, and 3′α, cleavages, respectively) appeared with almost the same intensities, i.e., 3′ε ≈ 3α > 3′α (Figure 1b,d, see also Figure S3). It must be noted that the full extent of the 3′ε cleavage cannot be determined because the 3′α cleavage, and the consecutive 3′ε and 3′α cleavages result in the same fragment ion at *m*/*z* 1262; thus, a portion of the 3′ε cleavage remains undetectable. Interestingly, the product ion (*m*/*z* 1472) formed by the 3α cleavage had a higher appearance maximum and energy range (up to 120 eV) compared to the other product ions resulting from the 2′ε, 3′ε, and 3′α cleavages (those decomposed at 100–110 eV). The maximum of the ERMS curve of the 3α cleavage was at 90 eV (SY_P1_ ≈ 10%), while that of the 2′ε, 3′ε, and 3′α cleavages was at 75 eV (SY_P1_ ≈ 35%). Any cleavage combination with the 3α occurred at energies about 10 eV higher than the other combinations (Figure 1f,h; see also Figure S3). Overall, it can be seen that the relative intensities of the fragment ions do not exceed 5–10% (except for the ion at *m*/*z* 1516), suggesting that there is high competition between the different fatty acyl cleavage sites.

### 2.2. Energy Dependence of the Intra- and Inter-Ring Fragmentations

**Figure 2:**
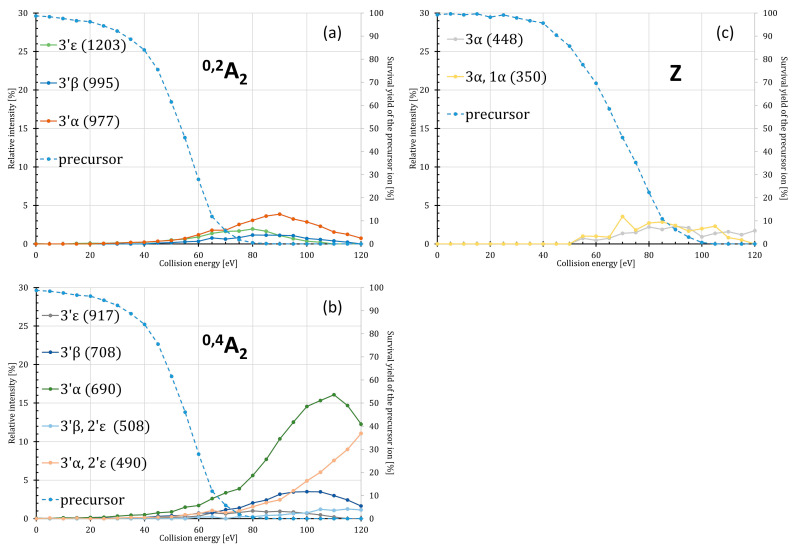
ERMS curves of the *E. coli*-type lipid A precursor ions, as well as fragment ions resulting from (**a**) the ^0,2^A_2_, (**b**) ^0,4^A_2_, and (**c**) Z cleavages combined with losses of the fatty acyl chains. The ERMS curves for the P4′ isomer are depicted on the left (**a**,**b**), while that for the P1 isomer is on the right (**c**). The numbers and Greek letters denote the cleavage sites and identify the fragment ions, the nominal ionic masses of which are indicated in parentheses; for exact masses of the ions and compositions of the neutral losses, see Table 2.

During low-energy CID conditions, the monophosphorylated lipid A species also undergo fragmentations along the diglucosamine backbone when exposed to CE higher than 50 eV (Figure 2 and Table 2). For P4′ isomers, the main processes are the intra-ring fragmentations giving ^0,2^A_2_- and ^0,4^A_2_-type ions; for P1 isomers, inter-ring fragmentation (involving cleavage of the glycosidic bond connecting the two glucosamine units) occurs, resulting in Z-type ions. The different types of ring cleavage products may help to identify the individual isomers; however, their formation at SY > 50% did, mostly, not exceed 1% (for the A-type ions) or 5% (for the Z-type ions). Exceptions were the ^0,4^A_2_ + 3′α (at *m*/*z* 690) and ^0,4^A_2_ + 3′α + 2′ε (at *m*/*z* 490) ions, of which intensities reached 17% and 12%, respectively. Unfortunately, even the relative intensities of Z ions diagnostic for P1 formation (*m*/*z* 448 and 350) did not exceed 4%.

### 2.3. Energy Dependence of the Meta- and Orthophosphate Ion Losses

**Figure 3:**
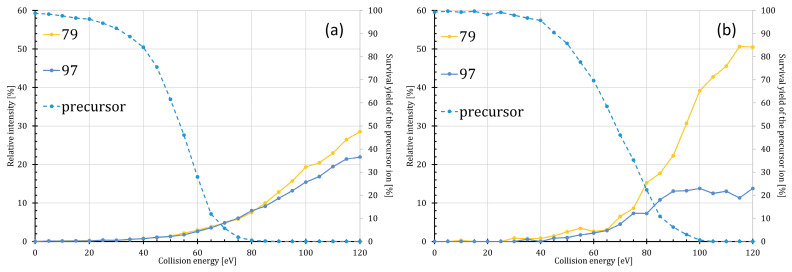
ERMS curves of the precursor and ortho- and metaphosphate ions (theoretical monoisotopic ion masses are 96.96962 u and 78.95905 u) formed during the CID of the (**a**) P4′ and (**b**) P1 variants of monophosphorylated hexaacylated *E. coli*-type lipid A species.

Cleavage of the phosphoester bond (either at position 4′ or 1) was also observed during the fragmentation of the monophosphorylated lipid A species. While the deprotonated metaphosphoric acid (monoisotopic ion mass 78.95905 u) and orthophosphoric acid (monoisotopic ion mass 96.96962 u) were detected, the complementary dephosphorylated lipid A ions were only observed for some Z-type ions. In the case of the P4′ isomer, the intensities of the *m*/*z* 97 and 79 ions were the same in the CE range of 0–80 eV (where SY_P4′_ > 0%), while above 80 eV the intensity of the *m*/*z* 79 ion increased slightly (Figure 3a). In contrast, for the P1 isomer, the *m*/*z* 79 ion was present in a significantly larger amount than *m*/*z* 97 (i.e., more than 20% difference in their relative intensities), when the CE was greater than 95 eV (SY_P1_ < 10%) (Figure 3b). This could be another signature of the presence of the P1 isomer in the sample.

### 2.4. Identification of Chimera Mass Spectra of Lipid A Isomers

In lipopolysaccharidomics studies, the unambiguous identification of chimera spectra is a major challenge. A careful examination of the ERMS curves of the phosphorylation isomers of lipid A gives a possibility of finding signature ions for each of the isomers. Comparing the CID fragmentation of the deprotonated P4′ and P1 isomers of the *E. coli*-type lipid A, we have observed that only certain routes lead to the formation of distinct ions for the isomers. Diagnostic cleavages for 1-phosphorylation are the cleavages of the 2′ε ester bond, its combinations with other ester bond cleavages (3′ε, 3′α, 3α), and the formation of Z ions. Diagnostic cleavages for 4′-phosphorylation are the 3′β bond cleavage, its combination with other bond cleavages (3α, 2′ε), and the formation of A_2_ ions. Unfortunately, among the above-mentioned diagnostic product ions, the 2′ε loss is the only one whose relative intensity is high enough (varying between 5% and 15%) in the energy range of 60–80 eV, while the other diagnostic ions’ abundances do not even reach 5%. All other product ions appear in both fragmentation pathways, albeit with slightly different formation energy ranges and quantities, vide supra. Generally, the P1 variant’s fragment ions are formed at higher energies than those of P4′. At high collision energies (where the precursor is completely decomposed), the P4′ and P1 isomers show differences in the cleavage of the amide-linked fatty acid at position 2, which occurs as the final step of the sequential release of fatty acids. For the P1 isomer, the amide-linked fatty acyl chain is released both as a ketene (2β) and an aldehyde (2δ); while in the case of the P4′ isomer, it is eliminated in the form of an amide (2α). Moreover, there is a clear difference between P4′ and P1 in the extent of the cleavage of the phosphoester bond at higher collision energies, vide supra.

For identification of a chimera mass spectrum, it is suggested to perform fragmentations at three different energy levels, such as at low (when 100% > SY > 50%), medium (when 50% > SY > 0%), and high energy levels (5–15 eV greater than CE needed to reach SY = 0%). In the case of a mixture of lipid A isomers, the characteristic collision energy (i.e., CCE, meaning the CE at SY 50%) can vary between two extrema (in our case, between 53 eV and 68 eV) depending on the molar ratio of the components. In this study, the difference in the apparent CCE of the P4′ and P1 isomers was 15 eV. In addition, the difference between the CE needed for the complete decomposition of the precursor ions can be even bigger (in our case, it was 85 eV for P4′ and 105 eV for P1). Consequently, we ought to restrict the three energy levels to a smaller range where the prerequisite would meet for both isomers. Thus, in the present study, the low CE level means 0–50 eV, medium means 70–85 eV, and high means 100–120 eV.

**Figure 4:**
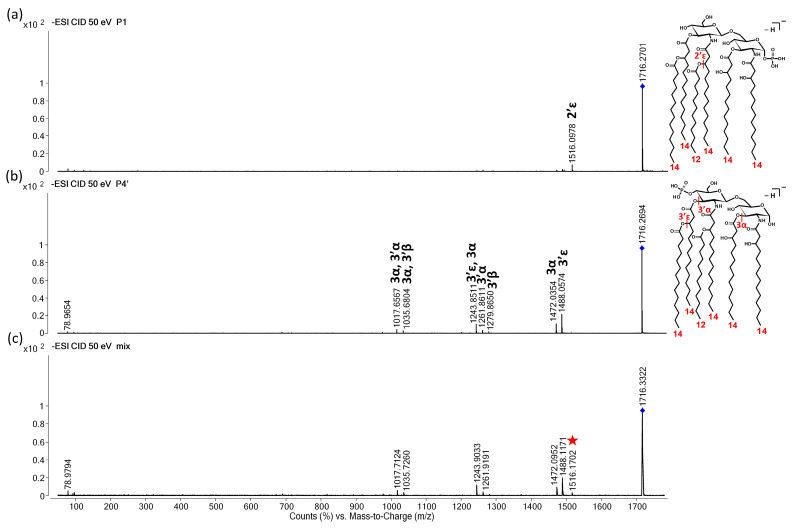
Representative CID mass spectra for the separated (**a**) P1 and (**b**) P4′ isomers, and (**c**) the direct-injected *E. coli* bacterial lipid A sample containing both isomers at an approximately 85:15 P4′-to-P1 molar ratio. Spectra were recorded in the low CE range (at 50 eV laboratory frame collision energy), where the precursor’s survival yield was higher than 50%. The numbers and Greek letters denote the cleavage sites and also identify the fragment ions. The structures of the separated isomers, along with the cleavage sites and number of carbon atoms within the fatty acyl chains, are depicted in the inserts. For exact masses of the ions and compositions of the neutral losses, see Table 1 and Table 2. The precursor ion is denoted by a rhombus in each mass spectrum. The diagnostic ion for P1 in the mixture is denoted by a star in the chimera mass spectrum.

**Figure 5:**
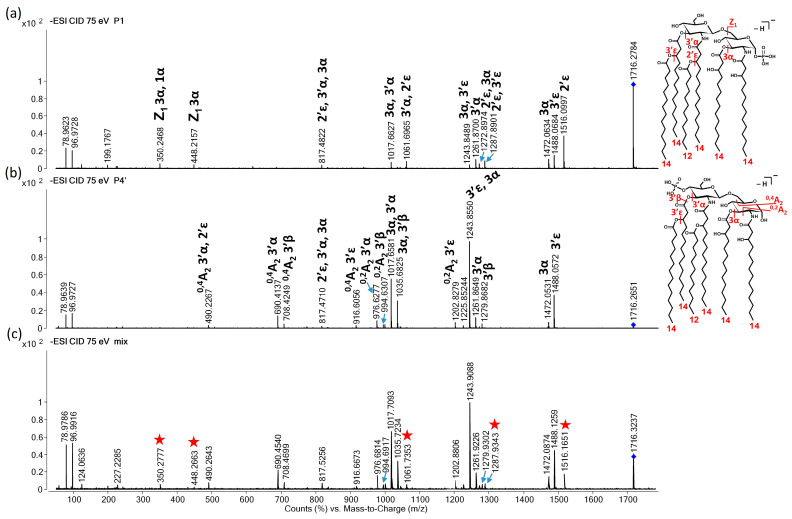
Representative CID mass spectra for the separated (**a**) P1 and (**b**) P4′ isomers, and (**c**) the direct-injected *E. coli* bacterial lipid A sample containing both isomers at an approximately 85:15 P4′-to-P1 molar ratio. Spectra were recorded in the medium CE range (at 75 eV laboratory frame collision energy), where the precursor’s survival yield was between 50% and 0%. The numbers and Greek letters denote the cleavage sites and also identify the fragment ions. The structures of the separated isomers, along with the cleavage sites and number of carbon atoms within the fatty acyl chains, are depicted in the inserts. For exact masses of the ions and compositions of the neutral losses, see Table 1 and Table 2. The precursor ion is denoted by a rhombus in each mass spectrum. The diagnostic ions for P1 in the mixture are denoted by stars in the chimera mass spectrum.

**Figure 6:**
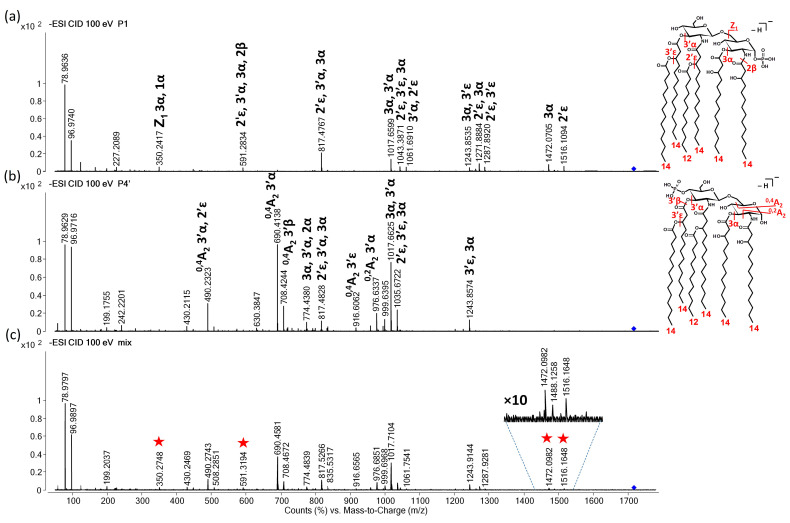
Representative CID mass spectra for the separated (**a**) P1 and (**b**) P4′ isomers, and (**c**) the direct-injected *E. coli* bacterial lipid A sample containing both isomers at an approximately 85:15 P4′-to-P1 molar ratio. Spectra were recorded in the high CE range (at 100 eV laboratory frame collision energy), where the precursor’s survival yield was 0%. The numbers and Greek letters denote the cleavage sites and also identify the fragment ions. The structures of the separated isomers, along with the cleavage sites and number of carbon atoms within the fatty acyl chains, are depicted in the inserts. For exact masses of the ions and compositions of the neutral losses, see Table 1 and Table 2. The precursor ion is denoted by a rhombus in each mass spectrum. The diagnostic ions for P1 in the mixture are denoted by stars in the chimera mass spectrum.

The negative-ion CID mass spectra of the P4′ and P1 isomers present in the in-house standard, i.e., an *E. coli* O83 extract, recorded at the three energy levels after a separation and without separation (i.e., after direct injection) are presented in Figure 4, Figure 5 and Figure 6. At the low energy level, the chimera mass spectrum highly resembled the CID mass spectrum of the P4′, and the only signature for the presence of the P1 isomer was the ion at *m*/*z* 1516, which was formed due to the 2′ε cleavage (Figure 4).

The chimera mass spectrum becomes more complex at the medium energy level since most of the fragmentation processes are ongoing in this range (Figure 5). The chimera mass spectrum recorded for the mixture of isomers (Figure 5c) still resembles the CID mass spectrum of the pure P4′ isomer (Figure 5b). Meanwhile, the diagnostic ion of P1 at *m*/*z* 1516 (2′ε cleavage) is accompanied by four other ions, such as ions at *m*/*z* 1288 (2′ε, 3′ε combined cleavages), 1062 (2′ε, 3′α), 448 (Z_1_, 3α), and 350 (either B_1_, 3′α, 2′ε or Z_1_, 3α, 1α combined cleavages).

At high energy levels (Figure 6), the fragmentation processes of the two isomers occur at different stages. For the P4′ lipid A, the first generation of ions (at the highest *m*/*z* range) is missing (Figure 6b); meanwhile, for the P1, all generations of the product ions (specifically those associated with the 3α cleavage) can still be detected (i.e., ions at *m*/*z* 1472, 1272, 1244, 1044, 1018, 817) (Figure 6a). Thus, in the chimera mass spectrum, the ion at *m*/*z* 1472 (the result of the 3α cleavage alone) clearly indicates the presence of P1 in the mixture (Figure 6c). The diagnostic ions of P1 can be detected with low intensity, such as at *m*/*z* 1516, 1288, 1062, 350, and also a new one at *m*/*z* 591 (which corresponds to the combined cleavages of 2′ε, 3′α, 3α, 2β). Moreover, the significantly more abundant metaphosphate ion (*m*/*z* 79) compared to the orthophosphate ion (*m*/*z* 97) also indicates the presence of the P1 isomer.

In summary, from the analysis of the hexaacylated *E. coli*-type P4′ and P1 variants, we can postulate rules about the structural analysis of lipid A species bearing an amide-linked 3-acyloxy-acyl chain at the 2′ position. To identify that we have a mixture of phosphorylation isomers, we need to perform fragmentations on at least two energy levels, one below and one above the collision energy of SY_50%_ of the precursor (i.e., low and medium, or low and high CE levels). If the ion assumed to be the result of the 2′ε cleavage is detectable at the low or medium energy level, this likely indicates that both P4′ and P1 isomers are present in the sample. Then, if the intensity ratio of the fragments observed at the highest *m*/*z* region (*c.a.* 200–250 u smaller than the precursor, i.e., ions at *m*/*z* 1516, 1488, 1472 resulting from 2′ε, 3′ε, and 3α cleavages for *E. coli*-type lipid A) is significantly altered between the different energy levels (i.e., low and medium, or low and high), then this can also prove that the sample contains both phosphorylation isomers and the mass spectra have chimeric nature. The intensity ratio of the orthophosphate and metaphosphate ions also has diagnostic significance at the high energy level (where SY = 0%).

## 3. Conclusions

Sequential fatty acid losses play a major role in the negative-ion tandem mass spectrometry-based, de novo structure elucidation strategies of 4′-monophosphorylated lipid A species. Attention should be drawn to the fact that the presence of a minor 1-monophosphorylated species besides the major 4′-monophosphorylated one could only result in a single extra ion in the group of first-generation fragment ions (namely the 2′ε) in the CID mass spectrum recorded in a low collision energy experiment, which has no significant effect on the structural analysis of the major 4′-monophosphorylated component. Meanwhile, if the 1-monophosphorylated component is the major species, it causes false or misleading interpretation of the CID mass spectra. Thus, during the analysis of natural bacterial extracts, the first step should always be the determination of the phosphorylation site, consequently revealing whether it is a mixture or a pure sample. An ion pair (i.e., phosphate and dephosphorylated lipid A ions) from 1α or 4′α bond cleavage would give direct information about 1- or 4′-phosphorylation; unfortunately, none of these bond cleavages are significant in the negative ionization mode at low energy. Moreover, if these bonds are split, the charge would not be carried by the lipid A part, but by the counterion, i.e., the phosphate ion. In addition to the formation of the orthophosphate ion (*m*/*z* 97), the formation of the metaphosphate ion (*m*/*z* 79) can also be observed. At high energies, the ratio of these two ions (i.e., whether they have similar or different intensities) may indicate whether the analyte is pure P4′ or not. However, further studies may be needed to determine whether the acylation pattern of the backbone affects the orthophosphate-metaphosphate ratio. It would be more straightforward to use the positive ionization mode for this purpose (i.e., for determination of the phosphorylation site).

At medium and high collision energies, there are more signature ions for the P1 isomers of the hexaacylated *E. coli*-type lipid A (i.e., 2′ε and its combinations with 3′ε and 3′α, and Z-type ions). However, it would not be easy to identify them for another lipid A species with an unknown acylation pattern using negative-ion tandem mass spectrometry. Therefore, we recommend using at least two energies for fragmentation. Thus, set the CID energy to two levels where the survival yield of the precursor is greater than 50% and where it is close to 0%. Then, if the intensity ratio of the ions at the highest *m*/*z* range (*c.a.* 200–250 u smaller than the precursor, i.e., 2′ε, 3′ε, and 3α cleavages) changes significantly between the multi-energy level measurements, it indicates the presence of 1-phosphorylated lipid A. Further investigations may also be required to determine the effects of different acylation patterns (other than hexaacylated *E. coli*-like) on the structure elucidation strategy of 1-phosphorylated lipid A molecules. There is a historical bias towards monophosphorylated hexaacylated *E. coli*-type lipid A variants published in the literature due to the previously unrevealed limitations of direct MS/MS approaches in the analysis of constitutional lipid A isomers and the resultant chimera mass spectra.

## 4. Materials and Methods

### 4.1. Chemicals and Reagents

LC-MS grade methanol, isopropyl alcohol, water, acetic acid (eluent additive for LC-MS), and HPLC grade dichloromethane were purchased from Honeywell (Seelze, Germany). HPLC grade ammonium hydroxide (32% *m*/*m*) was obtained from VWR (Fontenay-sous-Bois, France). Synthetic monophosphoryl lipid A-504 (PHAD-504) was purchased from Sigma-Aldrich, the exclusive Avanti Polar Lipids provider (Alabaster, AL, USA) in Hungary. About 0.1 mg of the standard was dissolved in 75 μL of methanol and 25 μL of dichloromethane.

### 4.2. Lipid A Isolation and Sample Preparation

*Escherichia coli* O83 strain was cultured at 37 °C in a laboratory fermenter on Mueller-Hinton broth at pH 7.2 and then collected by centrifugation. The bacterial lipopolysaccharides were extracted from acetone-dried organisms using the hot phenol/water procedure [39] and then lyophilized. Lipid A was released from the lipopolysaccharide by mild acid hydrolysis with 1% (*v*/*v*) acetic acid (pH 3.9) at 100 °C for 1 h, then the solution was centrifuged (8000× *g*, 4 °C, 20 min). The sediment was washed four times with distilled water and then lyophilized. The sample was dissolved in methanol/dichloromethane (3:1, *v*/*v*) and filtered through a 0.22 μm pore size filter.

### 4.3. High-Pressure Liquid Chromatography Coupled with Electrospray Ionization-Quadrupole-Time-of-Flight Tandem Mass Spectrometry

The energy-resolved mass spectrometry (ERMS) measurements were carried out with an Infinity 1290 UHPLC system (Agilent Technologies, Waldbronn, Germany) coupled with a 6530 Accurate Mass Q-TOF mass spectrometer (Agilent Technologies, Singapore), which applies a low-energy beam-type CID fragmentation method. The UHPLC-MS system was controlled, and the data was evaluated by Agilent MassHunter B.08.00 software. The chromatographic separation was performed using a Cortecs™ UPLC™ C18 column (150 mm × 2.1 mm, 1.6 μm particle size, Waters). The mobile phase A was composed of methanol/water (95:5, *v*/*v*) with 0.22% (*v*/*v*) NH_3_ and 0.1% (*v*/*v*) acetic acid, while the mobile phase B consisted of isopropyl alcohol with 0.22% (*v*/*v*) NH_3_ and 0.1% (*v*/*v*) acetic acid. Gradient elution and column conditioning were performed as follows: 0.00 min 0% B, 40.00 min 70% B, 40.01 min 90% B, 50.00 min 90% B, 50.01 min 0% B, and 60.00 min 0% B. The flow rate and the temperature were set at 0.25 mL/min and 45 °C, respectively. The injection volume was 3 μL. Flow injection was applied for direct ERMS measurements with a 0.2 mL/min flow rate of 55% B eluent. The Agilent Jet Stream ion source was operated using the following conditions: the pressure of nebulizing gas (N_2_) was 30 psig; the temperature and flow rate of drying gas (N_2_) were 300 °C and 8 L/min, respectively; temperature and flow rate of sheath gas were 350 °C and 11 L/min, respectively. The capillary voltage was set to 3 kV, the nozzle voltage to 1 kV, the fragmentor potential to 100 V, and the skimmer potential to 65 V. Negative-ion mass spectra of the column eluate were recorded in the range of *m*/*z* 1000–2000 at a measuring frequency of 5000 transients/s and a detection frequency of 2 GHz. For tandem mass spectrometry measurements, the following parameters were used: mass range, *m*/*z* 50–2000; acquisition rate, 500 ms/scan; quadrupole isolation width, 1.3 *m*/*z*; maximum precursor ions/cycle, 1; precursor ion active charge state, 1. For the ERMS analysis, CID mass spectra were recorded at various collision energies between 0 and 120 eV, with 5 eV increments. Since the exact collision energy depends on the type and manufacturer of the instrument and may change over time, we also provide the amount of precursor ion in the form of survival yield (SY) as a reference in addition to the laboratory frame collision energy.

### 4.4. ERMS Data Evaluation

Energy-resolved breakdown curves (i.e., plots of the relative intensities of peaks as a function of laboratory frame collision energy) were constructed from the recorded MS/MS mass spectra. Before generating the breakdown curves, the mass spectral data were manually filtered, meaning that isotopic peaks and signals corresponding to noise were removed from the mass list. The relative intensity of each fragment ion was calculated by dividing the intensity of the corresponding fragment ion by the sum of the intensities of all ions, including the intensity of the precursor ion remaining after fragmentation. The survival yield of the precursor (SY) was also calculated as the ratio of the intensity of the precursor ion to the sum of intensities of the precursor and fragment ions. The normalized breakdown diagrams were obtained by plotting the normalized intensity of the product ion of interest against the laboratory frame collision energy. The ion abundances were normalized to the highest intensity observed over the full, i.e., 0–120 eV, collision energy range.

## Figures and Tables

**Table 1:** Fatty acyl chain losses from the deprotonated monophosphorylated hexaacylated *E. coli*-type lipid A molecules. The monoisotopic mass of the precursor ion (C_94_H_176_N_2_O_22_P^−1^) is 1716.2458 u. The distribution of the six fatty acids on the backbone of the intact precursor is as follows: N2: C14:0(3-OH), O3: C14:0(3-OH), N2′: C14:0(3-O-C12:0), O3′: C14:0(3-O-C14:0). The cleavage site is identified by a number and a Greek letter [35], where the number indicates the carbon atom of the diglucosamine backbone and the Greek letter indicates the distance of the cleaved bond from the given carbon atom.

Cleavage Type ^#^	Elemental Composition of the Loss	Cleavage Site(s)	Monoisotopic Molecular Mass of the Loss	Monoisotopic Ion Mass of the Product Ion
**One acyl chain**				
C12:0	C_12_H_24_O_2_	2′ε	200.1776	1516.0682 *
C14:0	C_14_H_28_O_2_	3′ε	228.2089	1488.0369
C14:0(3-OH)	C_14_H_28_O_3_	3α	244.2038	1472.0419
**Two acyl chains**				
C12:0 and C14:0	C_26_H_52_O_4_	2′ε, 3′ε	428.3866	1287.8592 *
C14:0(3-O-C14:0)_ketene_	C_28_H_52_O_3_	3′β	436.3916	1279.8541 **
C12:0 and C14:0(3-OH)	C_26_H_52_O_5_	2′ε, 3α	444.3815	1271.8643
C14:0(3-O-C14:0)	C_28_H_54_O_4_	3′α	454.4022	1261.8436
C14:0 and C14:0(3-OH)	C_28_H_56_O_5_	3′ε, 3α	472.4128	1243.8330
**Three acyl chains**				
C12:0 and C14:0(3-O-C14:0)	C_40_H_78_O_6_	2′ε, 3′α	654.5798	1061.6659 *
C12:0, C14:0, and C14:0(3-OH)	C_40_H_80_O_7_	2′ε, 3′ε, 3α	672.5904	1043.6554
C14:0(3-OH) and C14:0(3-O-C14:0)_ketene_	C_42_H_80_O_6_	3α, 3′β	680.5955	1035.6503 **
C14:0(3-OH) and C14:0(3-O-C14:0)	C_42_H_82_O_7_	3α, 3′α	698.6061	1017.6397
**Four acyl chains**				
C12:0, C14:0(3-OH), and C14:0(3-O-C14:0)_ketene_	C_54_H_104_O_8_	2′ε, 3′β, 3α	880.7731	835.4727 **
C12:0, C14:0(3-OH), and C14:0(3-O-C14:0)	C_54_H_106_O_9_	2′ε, 3′α, 3α	898.7837	817.4621
C14:0(3-OH), C14:0(3-O-C14:0), and C14:0(3-OH)_amide_	C_56_H_111_N_1_O_9_	3α, 3′α, 2α	941.8259	774.4199 **
**Five acyl chains**				
C12:0, C14:0(3-OH), C14:0(3-O-C14:0), and C12:0_aldehyde_	C_66_H_130_O_10_	2′ε, 3′α, 3α, 2δ	1082.9664	633.2794 *
C12:0, C14:0(3-OH), C14:0(3-O-C14:0), and C14:0(3-OH)ketene	C_68_H_132_O_11_	2′ε, 3′α, 3α, 2β	1124.9770	591.2688 *
C14:0(3-OH), C14:0(3-O-C14:0), C14:0(3-OH)_amide_, and C12:0	C_68_H_135_N_1_O_11_	3α, 3′α, 2α, 2′ε	1142.0035	574.2423 **

^#^ The cleavages are grouped according to the number of the fatty acyl chain(s) present in the neutral loss. * The fragment ion was detected only in the case of the P1 isomer. ** The fragment ion was detected only in the case of the P4′ isomer.

**Table 2:** Intra- and inter-ring cleavages accompanied by fatty acyl chain losses from the deprotonated monophosphorylated hexaacylated *E. coli*-type lipid A species. The nomenclature proposed by Domon and Costello [36] was used for denoting the intra- (corresponding to ^0,2^A_2_ and ^0,2^A_2_-type ions) and inter-ring (corresponding to B- and Z-type ions) cleavages. The cleavages of the side chains were identified by a number and a Greek letter [35], where the number indicates the carbon atom of the diglucosamine backbone and the Greek letter indicates the distance of the cleaved bond from the given carbon atom.

Cleavage Type ^#^	Elemental Composition of the Loss	Cleavage Site(s)	Monoisotopic Molecular Mass of the Loss	Monoisotopic Ion Mass of the Product Ion
**^0,2^A_2_-type ions for P4′**	C_16_H_31_NO_3_	^0,2^A_2_	285.2304	1431.0154 *
C14:0	C_30_H_59_NO_5_	^0,2^A_2_, 3′ε	513.4393	1202.8065
C14:0(3-O-C14:0)_ketene_	C_44_H_83_NO_6_	^0,2^A_2_, 3′β	721.6220	994.6237
C14:0(3-O-C14:0)	C_44_H_85_NO_7_	^0,2^A_2_, 3′α	739.6326	976.6132
**^0,4^A_2_-type ions for P4′**	C_32_H_61_NO_7_	^0,4^A_2_	571.4448	1144.8010 *
C14:0	C_46_H_89_NO_9_	^0,4^A_2_, 3′ε	799.6537	916.5921
C14:0(3-O-C14:0)_ketene_	C_60_H_113_NO_10_	^0,4^A_2_, 3′β	1007.8364	708.4093
C14:0(3-O-C14:0)	C_60_H_115_NO_11_	^0,4^A_2_, 3′α	1025.8470	690.3988
C12:0 and C14:0(3-O-C14:0)_ketene_	C_72_H_137_NO_12_	^0,4^A_2_, 3′β, 2′ε	1208.0141	508.2317
C12:0 and C14:0(3-O-C14:0)	C_72_H_139_NO_13_	^0,4^A_2_, 3′α, 2′ε	1226.0246	490.2211
**B-type ions for P4′**	C_34_H_65_NO_9_	B_1_	631.4659	1084.7799 *
C14:0(3-O-C14:0)_ketene_	C_62_H_117_NO_12_	B_1_, 3′β	1067.8576	648.3882
C14:0(3-O-C14:0)	C_62_H_119_NO_13_	B_1_, 3′α	1085.8681	630.3776
C12:0 and C14:0(3-O-C14:0)_ketene_	C_74_H_141_NO_14_	B_1_, 3′β, 2′ε	1268.0352	448.2106
C12:0 and C14:0(3-O-C14:0)	C_74_H_143_NO_15_	B_1_, 3′α, 2′ε	1286.0458	430.2000
**Z-type ions for P1**	C_60_H_113_NO_11_	Z_1_	1023.83136	692.4144 ^#^
C14:0(3-OH)	C_74_H_141_NO_14_	Z_1_, 3α	1268.0352	448.2106
C14:0(3-OH) and H_3_PO_4_	C_74_H_144_NO_18_P	Z_1_, 3α, 1α	1366.0121	350.2337

^#^ The cleavages are grouped separately according to the type of ring cleavages for the P4′ and P1 isomers. * Non-detected intact intra- or inter-ring fragment ions.

## Data Availability

Data is contained within the article or Supplementary Materials.

## Supplementary Materials

The following supporting information can be downloaded at: https://www.mdpi.com/article/10.3390/toxins16070322/s1, Figure S1: Extracted ion chromatogram for the monophosphorylated hexaacylated *E. coli*-type lipid A species (*m*/*z* 1716.24) with known structures; Figure S2: Structural confirmation of the 1-phosphorylated (P1) and 4′-phosphorylated (P4′) isomers of the *E. coli*-type hexaacylated lipid A by positive-ion ESI-QTOF MS/MS; Figure S3: Normalized representation of energy-resolved mass spectrometry curves of precursor ions, as well as fragment ions resulting from the consecutive and competitive losses of fatty acyl chains; Figure S4: ERMS curves of the precursor and fragment ions of the PHAD-504 standard with error bars and of the P4′ isomer from *E. coli* O83 bacterium; Figure S5: Structures of the phosphopositional isomers with the depiction of all the cleavage sites observed during the energy-resolved mass spectrometry experiments.
