# Supplementary material for: Identification of a Chimera Mass Spectrum of Isomeric Lipid A Species Using Negative Ion Tandem Mass Spectrometry
Source: Toxins (Basel). 2024 Jul 18;16(7):322. doi: 10.3390/toxins16070322 (PMC11281664; doi:10.3390/toxins16070322)
Supplement: Supplementary file 1 [file toxins-16-00322-s001.zip › toxins-3088984-supplementary.pdf]

Supplementary Information

Identification of a Chimera Mass Spectrum of Isomeric Lipid A Species Using Negative Ion Tandem Mass Spectrometry

Ágnes Dörnyei, Anikó Kilár and Viktor Sándor

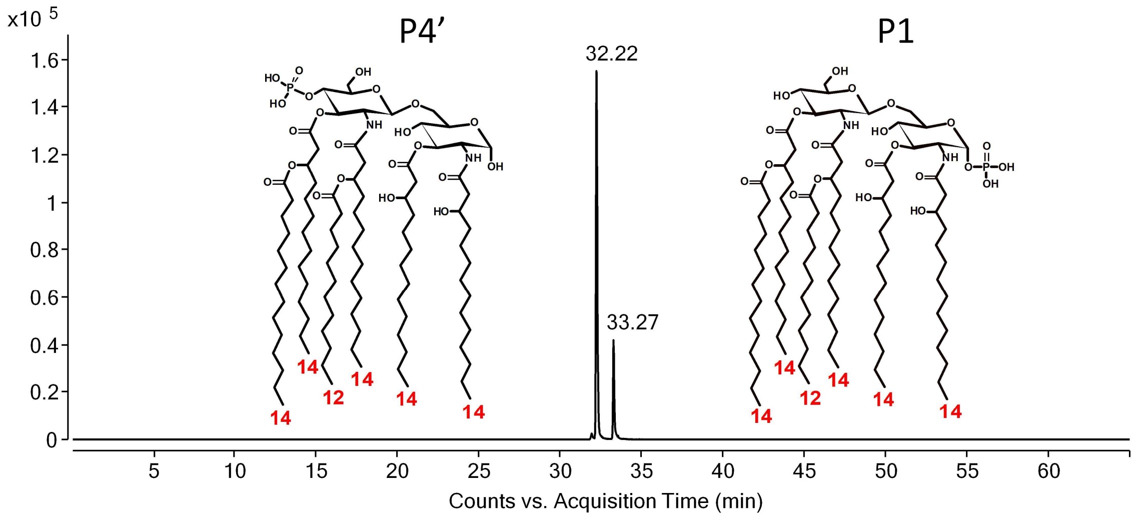

Figure S1 Extracted ion chromatogram for the monophosphorylated hexaacylated *E. coli*-type lipid A species. Two isomers (P4′ and P1) – differing only in the position of the phosphate group – were baseline separated and then detected in the negative ionization mode by HPLC-ESI-QTOF MS analysis. The theoretical monoisotopic mass of the deprotonated lipid A molecule is 1716.2458 u.

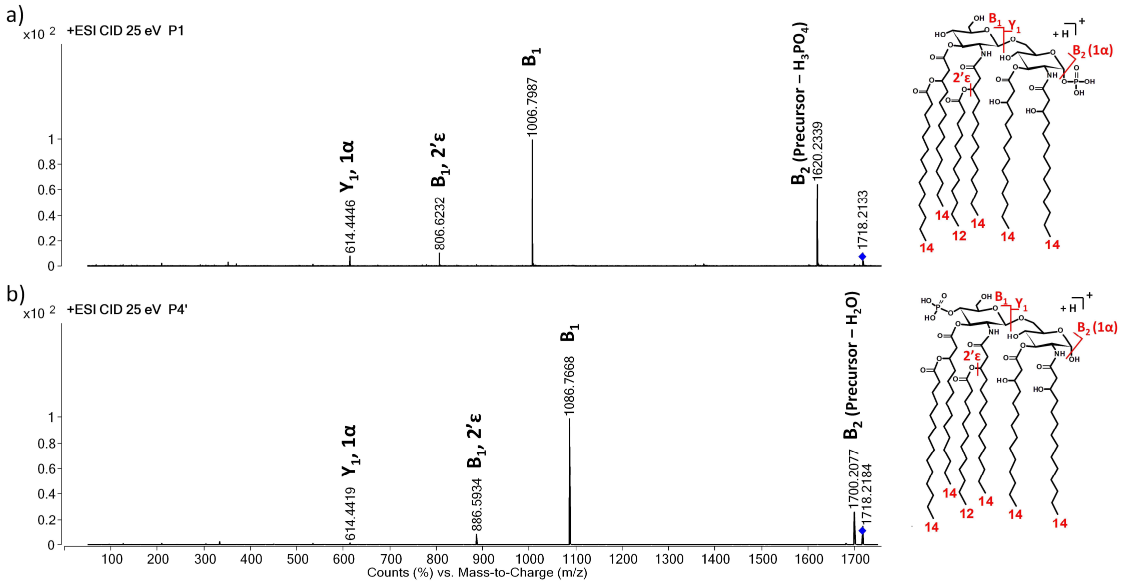

Figure S2 Structural confirmation of the (a) 1-phosphorylated (P1) and (b) 4′-phosphorylated (P4′) isomers of the *E. coli*-type hexaacylated lipid A. The analyses were carried out on completely separated isomers by HPLC-ESI-QTOF MS/MS in positive ionization mode. The ions observed in the mass spectra are explained by presenting the structure of the isomers and marking the cleavage sites. The theoretical monoisotopic mass of the protonated lipid A molecule is 1718.2603 u.

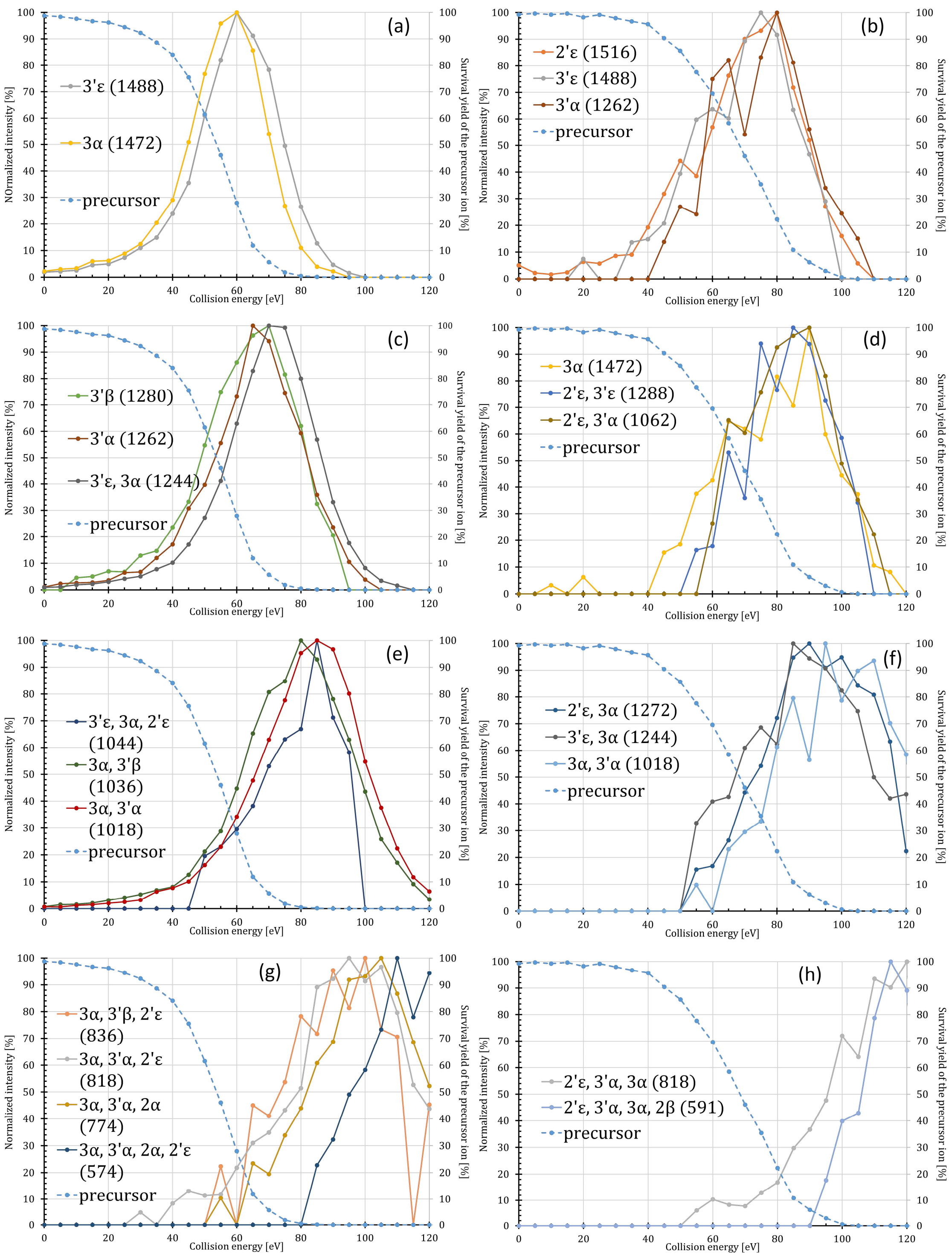

Figure S3 Normalized ERMS curves of the lipid A precursor and fragment ions (those of the P4′ isomer in the left and those of the P1 isomer are in the right column) resulting from the consecutive and competitive losses of fatty acyl chains. The numbers and Greek letters denote the cleavage sites and are, therefore, also used to identify the fragment ion type; the nominal ionic masses are denoted in parentheses. For exact masses of the ions and compositions of the neutral losses, see Table 1.

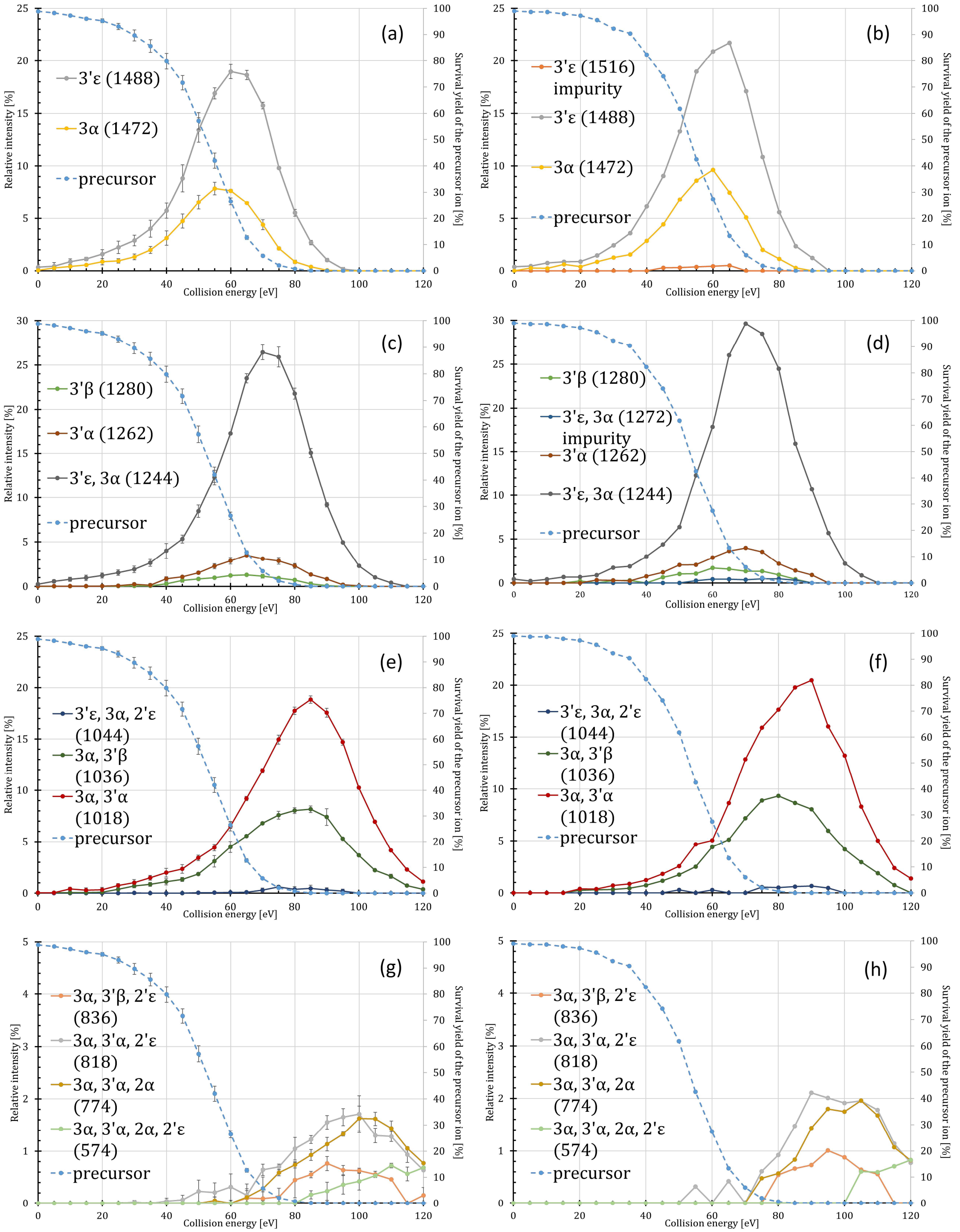

Figure S4 ERMS curves of the precursor and fragment ions of the PHAD-504 standard (a, c, e, g) with error bars (number of repetitions: 3) and of the P4′ isomer from *E. coli* O83 bacterium (b, d, f, h). For exact masses of the ions and compositions of the neutral losses, see Table 1. The low-intensity ions at *m/z* 1516 (b) and 1272 (d), which occurred for the bacterial P4′ isomer, most probably are derived from a minor acyl group-based isomer present in approximately 2% of the precursor ions in the bacterial sample.

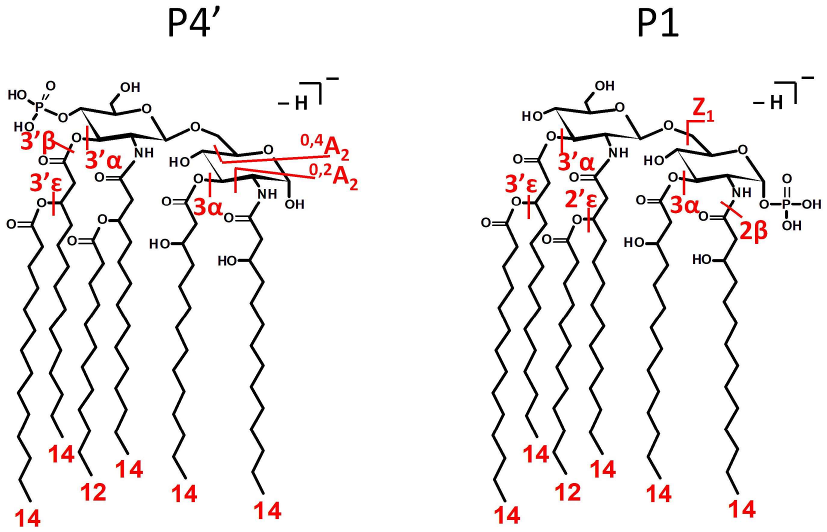

Figure S5 Structures of the phosphopositional isomers with the depiction of all the cleavage sites observed during the energy-resolved mass spectrometry experiments
